# Biogenic α-Fe_2_O_3_ nanoparticles from *Sorghum bicolor* leaf extracts and assessment of the anticancer and antioxidant properties

**DOI:** 10.1186/s11671-025-04281-z

**Published:** 2025-07-15

**Authors:** Lerato D. Msimango, Mercy C. Ogwuegbu, Doctor M. N. Mthiyane, Damian C. Onwudiwe

**Affiliations:** 1 Department of Animal Science, Faculty of Natural and Agricultural Science, North-West University, Mmabatho, 2035 South Africa; 2 Food Security and Safety Focus Area, Faculty of Natural and Agricultural Sciences, North-West University, Mmabatho, 2735 South Africa; 3 Department of Animal Science, Faculty of Agriculture, University of Nigeria, Nsukka, 410001 Enugu State, Nigeria; 4 Materials Science Innovation and Modelling (MaSIM), Faculty of Natural and Agricultural Sciences, North-West University, Mmabatho, South Africa; 5 Department of Chemistry, School of Physical and Chemical Sciences, Faculty of Natural and Agricultural Science, North-West University (Mafikeng Campus), Private Bag X2046, Mmabatho, South Africa

**Keywords:** Green chemistry, Iron oxide, Biosynthesis, Cytotoxicity, Antioxidants

## Abstract

The synthesis of nanomaterials has recently shifted toward environmentally benign approaches that mitigate the drawbacks of conventional chemical methods. In this context, plant-mediated green synthesis offers a sustainable and versatile alternative for producing nanoparticles with unique physicochemical properties and diverse applications. This study presents the green synthesis of hematite iron oxide nanoparticles (α-Fe_2_O_3_ NPs) using aqueous leaf extracts of *Sorghum bicolor*. The resulting nanoparticles were characterized using X-ray diffraction (XRD), UV–visible spectroscopy, scanning electron microscopy (SEM), transmission electron microscopy (TEM), and energy-dispersive X-ray spectroscopy (EDX). XRD analysis confirmed the formation of a crystalline rhombohedral hematite phase with an average crystallite size of 46.8 nm. SEM and TEM images revealed predominantly spherical particles with evident agglomeration, while EDX analysis confirmed iron (Fe) and oxygen (O) as the primary elemental constituents. Antioxidant activity assessed via the 2,2-diphenyl-1-picrylhydrazyl (DPPH) assay showed a concentration-dependent radical scavenging effect, with higher α-Fe_2_O_3_ NP concentrations required to achieve 50% inhibition. Cytotoxicity studies on HeLa (cancer) and HEK293 (normal) cell lines indicated selective toxicity, with the nanoparticles preferentially affecting cancer cells while sparing healthy ones. Although the α-Fe_2_O_3_ NPs exhibited lower potency compared to the standard chemotherapeutic agent 5-fluorouracil, their concentration-dependent reduction in cell viability supports the hypothesis that cancer cells are particularly vulnerable to disruptions in iron homeostasis. This cost-effective and eco-friendly synthesis method underscores the potential of *Sorghum bicolor*-mediated α-Fe_2_O_3_ nanoparticles for future biomedical applications.

## Introduction

Metal oxide nanoparticles obtained through green synthesis are gaining greater acceptability over those obtained through traditional chemical synthesis. The use of plant extracts for the green synthesis of metal oxide nanoparticles offers several environmental benefits compared to traditional methods, including the use of non-toxic reagents, environmental friendliness, reduced energy consumption, and ease of synthesis [1, 2]. The phytochemicals present in plant extracts act as natural reducing and capping agents, facilitating nanoparticle formation under ambient or mild reaction conditions, which in turn reduces energy consumption compared to high-temperature or high-pressure conventional methods [3]. This approach promotes the use of renewable and biodegradable resources, aligning with the principles of green chemistry and circular economy [4]. It supports waste valorization, especially when agro-waste or non-edible plant parts are used, further enhancing the environmental sustainability of the process. This is mainly because of their phytochemicals, such as flavonoids, terpenoids, carotenoids, and polyphenols present in plant parts– peels, stems, leaves, and fruits that serve as reducing agents during the synthesis [5, 6]. The biocompatibility of nanoparticles obtained through this technique makes them important materials in biological applications such as drug delivery, disinfection, anticancer agents, and antioxidant [7, 8].

Furthermore, this eco-friendly approach aligns with the principles of green chemistry and enhances sustainability by utilizing renewable, biodegradable resources. Consequently, it contributes to cleaner production processes and supports the development of sustainable nanomaterials with lower environmental footprints [9, 10].

Although metal oxide nanoparticles could potentially induce oxidative stress, some nanoparticles exhibit excellent activity as anticancer and antioxidants, such as CeO_2_, ZnO, NiO, Co_3_O_4_, and ZnO–MgO–CaO [11–14]. As antioxidants, metal oxides mimic the activity of natural antioxidant enzymes by directly scavenging reactive oxygen species (ROS) such as superoxide radicals and hydrogen peroxide, making them suitable agents for therapeutic application against oxidative stress-related diseases [15, 16]. Interestingly, metal oxides can act as effective anticancer agents primarily by generating reactive oxygen species (ROS), inducing oxidative stress, triggering apoptosis, and interfering with cellular processes through the release of metal ions. Separately, they can also exhibit antibacterial properties by disrupting bacterial cell membranes and generating ROS. This makes them potential alternatives to traditional antibiotics in overcoming antimicrobial resistance [16, 17].

Among the several green-synthesized metal oxides that have been explored for biomedical applications [12–14], iron oxides have gained much interest because of their high biocompatibility, and unique physical characteristics such as low susceptibility to oxidation, long blood half-life, and flexible surface chemistry [18, 19]. Superparamagnetic iron oxide nanoparticles incorporated with mesoporous silica nanoparticles, with the surface functionalized by NH_2_-bonding have been used as oxaliplatin loading and colon cancer treatment [20]. Sabouri et al. [21] reported amino-functionalized core-shell magnetic iron oxide nanoparticles used for inhibiting cancer cells. They have also been used as potential nanocarriers in drug delivery systems [22].

The hematite phase (α-Fe_2_O_3_) has particularly gained much traction compared to other oxides because of its exceptional stability under ambient conditions, making it highly resistant to corrosion and degradation. It is also relatively cheap and abundantly available in nature, making it an ideal material for several applications [23–25]. The green synthesis of α-Fe_2_O_3_, therefore, provides a facile and less-toxic route to obtaining a potentially effective antioxidant and anticancer agent.

Herein, in this study we have explored the use of aqueous extracts of *Sorghum bicolor* as an anticancer and antioxidant agent. *Sorghum bicolor* is a flowering plant belonging to the Poaceae family. It possesses biomolecules with therapeutic, functional, and nutraceutical properties that have been explored in the treatment of different ailments [26, 27]. The presence of these functional biomolecules makes extracts from *Sorghum bicolor* suitable for the synthesis of nanoparticles [3, 28, 29]. *Sorghum bicolor* leaf extract was chosen for the synthesis of α-Fe_2_O_3_ nanoparticles due to its rich phytochemical composition, which includes polyphenols, flavonoids, and alkaloids. These biomolecules facilitate the reduction of ferric ions and act as natural mediating agents, promoting the formation of stable nanoparticles. Additionally, *Sorghum bicolor* is an easily accessible, renewable, and eco-friendly resource, aligning with the principles of green chemistry and sustainable nanomaterial synthesis. Previous reports have also demonstrated the plant’s efficacy in the biosynthesis of metal-based nanoparticles, further supporting its selection for this study. The potential anticancer and antioxidant activities of this nanomaterial could lead to the production of materials that could serve as alternatives to traditional drugs.

## Materials and methods

### Plant collection and reagents

*Sorghum bicolor* leaf was harvested from the North-West University research farm at Mafikeng Campus. High purity (99%) ferrous sulfate heptahydrate (FeSO_4_·7H_2_O) and sodium hydroxide (NaOH) were purchased from Merck, RSA, and used as received.

### Preparation of aqueous plant extract

**Fig. 1:**
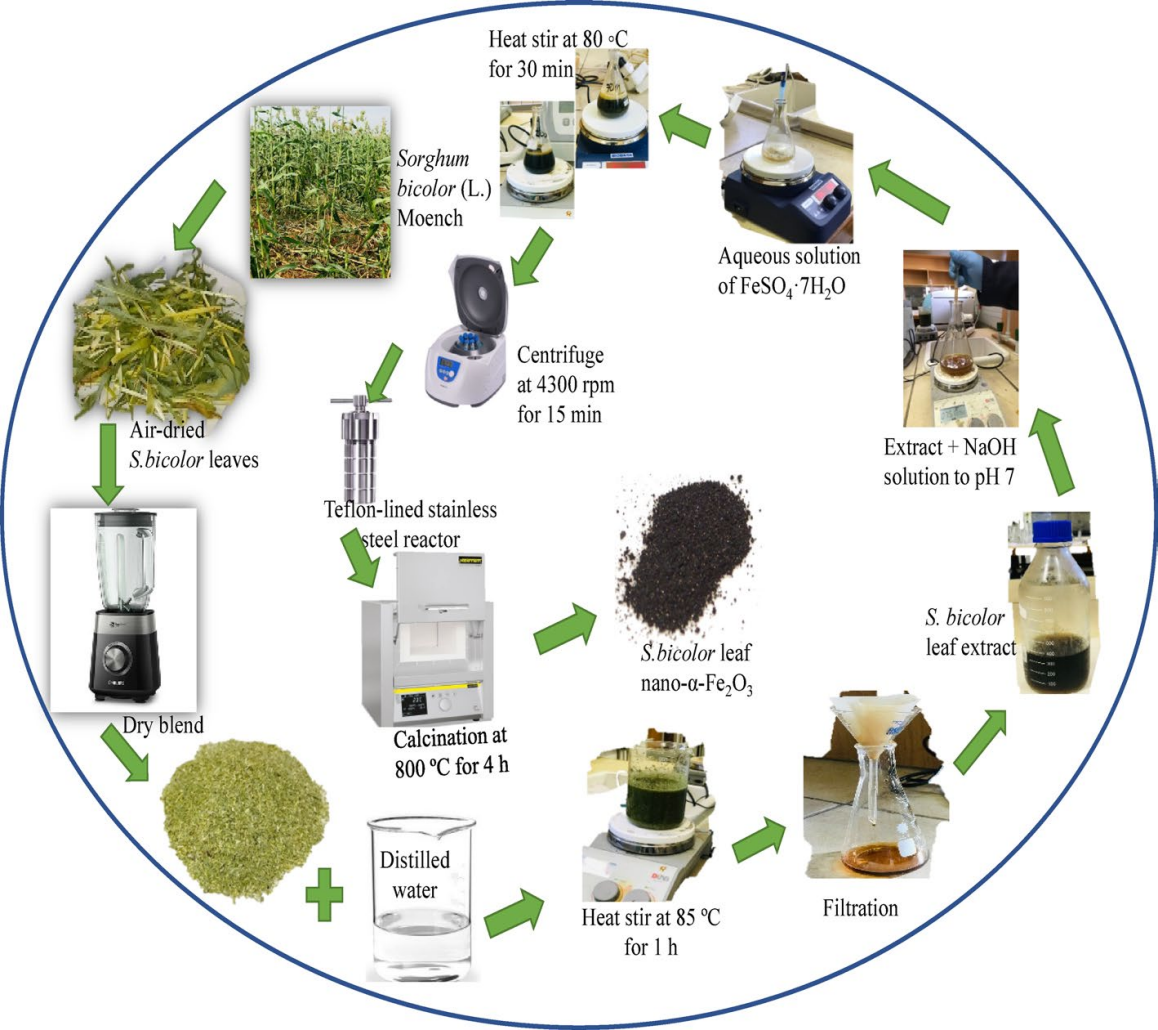
The schematic representation of the preparation of aqueous extract of *Sorghum bicolor* leaf and the green synthesis of α-Fe_2_O_3_ -NPs

The fresh *Sorghum bicolor* leaves were harvested, chopped into smaller pieces, and dried at room temperature for 28 days. The dried leaves were pulverised using a dry blender, and thereafter about 8 g of the powder was mixed with 100 mL of distilled water, followed with heating at 85 °C for 1 h while vigorously stirring. The mixture was allowed to cool to room temperature, filtered using no. 1 Whatman filter paper, and kept for further use [30]. Figure 1 presents the schematic steps for the extraction and synthesis processes.

### Qualitative phytochemical screening of *Sorghum bicolor leaves*

The presence of various phytochemical constituents in *Sorghum bicolor* leaves was assessed using procedures as outlined in previous reports in the literature [31, 32].

#### Saponin test

Approximately 0.3 g of the leaf powder was mixed with 30 mL of distilled water, heated in a water bath for 10 min, and then filtered through Whatman No. 42 filter paper (125 mm). To ensure the stability of the resulting foam, about 10 mL of the filtrate was mixed with 5 mL of distilled water and vigorously shaken. Three drops of olive oil were then added to the resulting froth, and the mixture was stirred vigorously to observe the formation of a stable emulsion, indicating the presence of saponins.

#### Terpenoid test

About 0.3 g portion of the leaf powder was soaked in 30 mL of distilled water and left to stand for 2 h. Subsequently, 5 mL of the extract was mixed with 2 mL of chloroform and 3 mL of conc. sulphuric acid to form a separate layer. The appearance of a reddish-brown coloration at the interface was indicative of terpenoids.

#### Flavonoid test

About 0.3 g portion of the leaf powder was soaked in 30 mL of distilled water, heated in a water bath for 2 h, and filtered using Whatman No. 42 filter paper (125 mm). To 10 mL of this aqueous filtrate, 5 mL of 1 M ammonia solution was added, followed by 5 mL of conc. sulphuric acid. A transient yellow coloration signified the presence of flavonoids.

#### Tannins test

A 0.3 g portion of *Sorghum bicolor* leaf powder was weighed and heated in 30 mL of distilled water using a water bath for 10 min. The mixture was then filtered, and to 5 mL of the resulting filtrate, three drops of 0.1% ferric chloride solution were added. The resulting colour was observed for a brownish-green or blue-black hue, indicating the presence of tannins.

### Synthesis of hematite α-Fe_2_O_3_ nanoparticles

α-Fe_2_O_3_ nanoparticles were prepared by following a previously reported procedure with slight modification [33]. In brief, 3.0 g of FeSO_4_·7H_2_O was dissolved in 100 mL distilled water and was heated to 80 ⁰C. While vigorously stirring the solution, 30 mL of the fresh aqueous extract of *Sorghum bicolor* leaf, whose pH has been adjusted to 10 using NaOH solution, was added dropwise. The resultant mixture was stirred for 30 min, and a caramel colour was observed. The final volume of the reaction solution was set to 70 mL and transferred to a Teflon-lined stainless-steel reactor (Autoclave) and heated to 145 °C for 2 h. The obtained particles were centrifuged at 4300 rpm for 20 min, rinsed twice with distilled water, and then followed with ethanol. The precipitate was transferred to a clean crucible and calcinated at 800 °C for 4 h, giving about 65% yield. The synthesized nanoparticles were kept for characterization.

### Characterization of α-Fe_2_O_3_-NPs

The X-ray diffraction (XRD) technique was used to analyse the crystalline structure of the nanoparticles (NPs), using a Bruker D8 Advanced diffractometer. Measurements were performed at room temperature over a 2θ diffraction angle range of 20° to 70°, with a step size of 0.05° and a counting time of 5 s per step, utilizing a Cu radiation source and Bragg-Brentano geometry. The UV-visible spectral analysis of α-Fe_2_O_3_ NPs was conducted in ethanol (as solvent) using a PROVE 300 with a resolution of 1 nm, across the wavelength range of 200 to 800 nm. Functional groups involved in the reduction process by the leaf extract were identified using a Cary 630 (Agilent, Palo Alto, USA) Fourier Transform Infrared (FTIR) spectroscopy, which was measured in the spectral range of 500–4000 cm^− 1^. The powdered leaf samples were used as the input material.

The external morphology of the nanoparticles was examined using scanning electron microscopy (SEM) (JSM 6510LV JEOL). Transmission electron microscopy (TEM) was utilized to determine the size and morphology of the NPs, using a JEOL-2100 TEM (100/120 kV) operating at 200 kV. For sample preparation, the NPs were dispersed in isopropanol and subjected to 10–15 min of sonication. Thereafter, a drop of the dispersion was placed onto a carbon grid, and the samples were analyzed after the solvent had evaporated. ImageJ software was used to determine the average particle size in the solid state.

### Cytotoxicity evaluation

The cytotoxic effects of α-Fe_2_O_3_-NPs were assessed based on the method described in [34], with some modifications. HeLa and HEK293 cell lines used for the assay were sourced from ATCC, Manassas, USA. Liver and kidney cell lines were grown in 25 cm² tissue culture flasks with a medium containing 10% foetal bovine serum, 100 µg/mL streptomycin, and 100 U/mL penicillin. For the MTT assay, 2.5 × 10² cells/well in 100 µL of DMEM were plated in a 96-well plate and incubated overnight at 37 °C. The nanoparticles were added at concentrations of 10, 25, 50, and 100 µg/mL, and the cells were incubated for 48 h at 37 °C. 5-fluorouracil (5-FU) served as the reference treatment. After 48 h, the medium was replaced with fresh media containing 10% MTT reagent, and the cells were incubated for an additional 4 h at 37 °C. Following incubation, the formazan crystals formed were dissolved in 100 µL of dimethyl sulfoxide (DMSO), and the absorbance was measured at 570 nm, with DMSO as the blank. The experiment was conducted in triplicate, and the average absorbance was calculated. Cell viability was determined using the Eq. (1).

1 $$\% \;{\text{Cell}}\;{\text{viability }} = {\text{ Treated}}\;{\text{cells/Untreated}}\;{\text{cells }} \times {\text{ 1}}00$$

### Antioxidant activity evaluation

The radical scavenging activity of the α-Fe_2_O_3_ -NPs was assessed using a modified version of the DPPH assay, based on the procedure outlined in [8], with some adjustments. To prepare the DPPH solution, about 12.8 mg of DPPH was dissolved in methanol (50 mL) to reach a conc. of 0.032 mM, and the solution was allowed to incubate in the dark for 30 min. Thereafter, α-Fe_2_O_3_ solutions at various concentrations (31.25, 62.5, 125, 250, 500, and 1000 ppm) were prepared in 1 mL of methanol. Then, 1 mL of the DPPH solution was introduced into each α-Fe_2_O_3_ solution, while the blank consisted of only the DPPH solution and methanol. Absorbance was recorded at 540 nm, with the control being the 0.032 mM DPPH solution. The experiment was performed in triplicate, and the percentage inhibition was determined using Eq. 2.

2 $$\% {\text{Inhibition }} = {\text{ }}\left( {{\text{A}}_{{\text{c}}} - {\text{A}}_{{\text{s}}} } \right){\text{ }}/{\text{A}}_{{\text{c}}} \; \times \;{\text{100}}\%$$

where (A_c_) is the absorbance of the control, and (A_s_) represents the sample’s absorbance.

## Results and discussion

### Synthesis mechanism

**Fig. 2:**
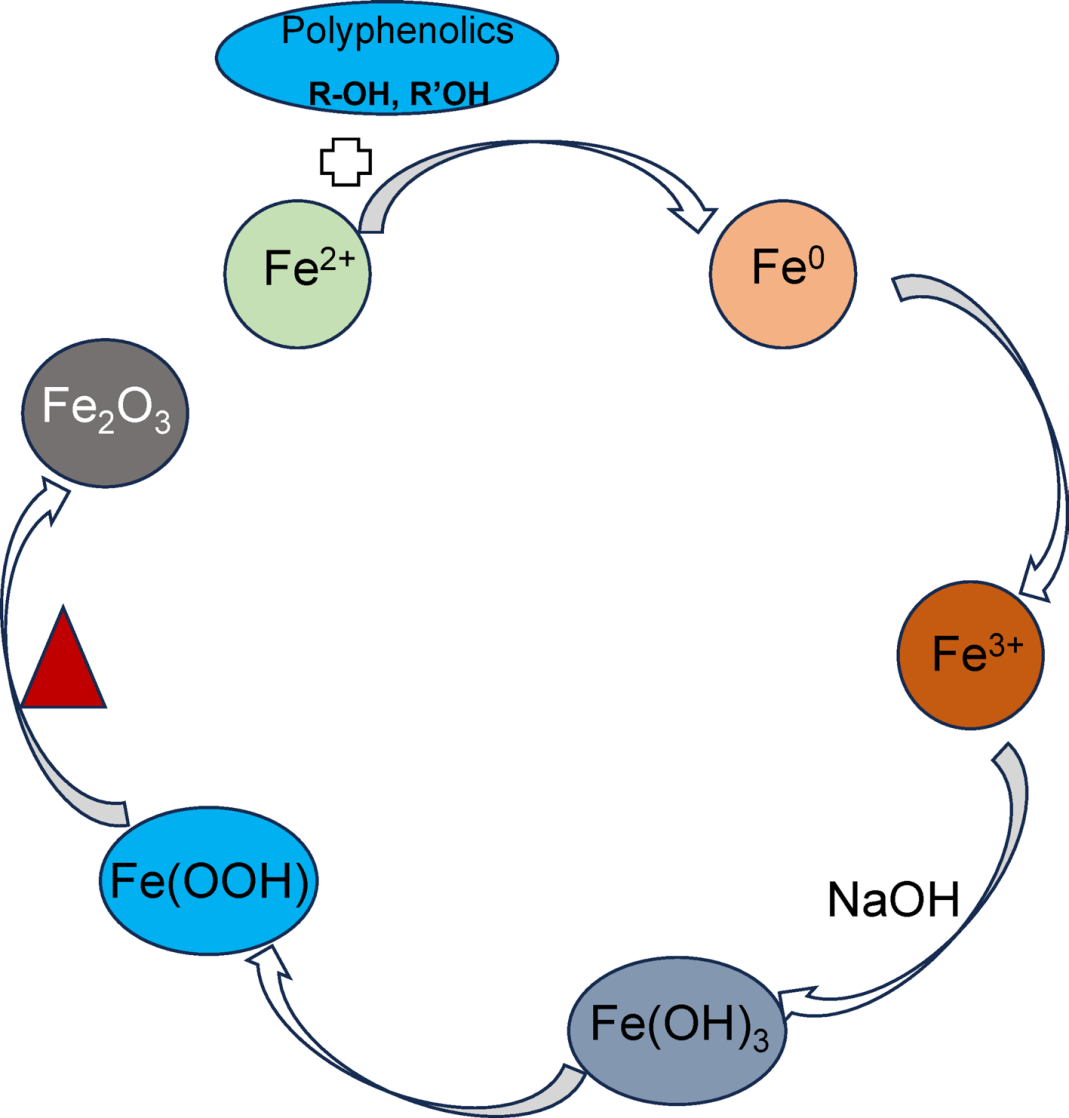
A plausible mechanism for formation of the α-Fe_2_O_3_ NPs using aqueous extract of *Sorghum bicolor* leaves

The phytochemical analysis of the leaf extract of *Sorghum bicolor* revealed the presence of bioactive compounds, including flavonoids, alkaloids, tannins, saponins, and triterpenes, as shown in Table 1, and similar to previous reports [35, 36]. These phytochemicals have the capacity to reduce metal ions in a metallic salt solution to their zero valent state. A plausible mechanism for the formation of the α-Fe_2_O_3_ NPs is presented in Fig. 2. In the first step of the process, the Fe^2+^ was reduced to Fe^0^ while the oxidized product of the polyphenolic compound was formed. The polyphenols present in plant extracts have been reported to have a high value of standard reduction potential (between 0.534 and 0.540 V) [37] that is enough to reduce metals such as iron (standard potential − 0.44 V [38]) to their respective nanoparticles [38, 39]. In the second step, due to the high instability of the Fe^0^, an immediate oxidation to Fe^3+^ occurred. This is a typical metal core-shell oxidation reaction, and similar processes have been reported [40]. Due to the presence of NaOH and adjustment of the pH to the basic medium, the formation of Fe(OH)_3_ occurred, subsequently forming Fe(OOH). The calcination process removed a water molecule and resulted to the hematite nanoparticles as presented in Eq. 3.

3 $${\text{2Fe}}\left( {{\text{OOH}}} \right) \longrightarrow ~\upalpha {\text{-Fe}}_{{\text{2}}} {\text{O}}_{{\text{3}}} + {\text{ H}}_{{\text{2}}} {\text{O}}$$

**Table 1:** Qualitative analysis of phytochemicals present in *Sorghum bicolor* leaves

Compounds	*M. azedarach* seed
Saponin	+
Terpenoids	++
Flavonoids	++
Tannins	++

Heavily present…………….++

Present………………………+

Absent……………………….−

### Fourier transform infrared (FTIR) analysis

**Fig. 3:**
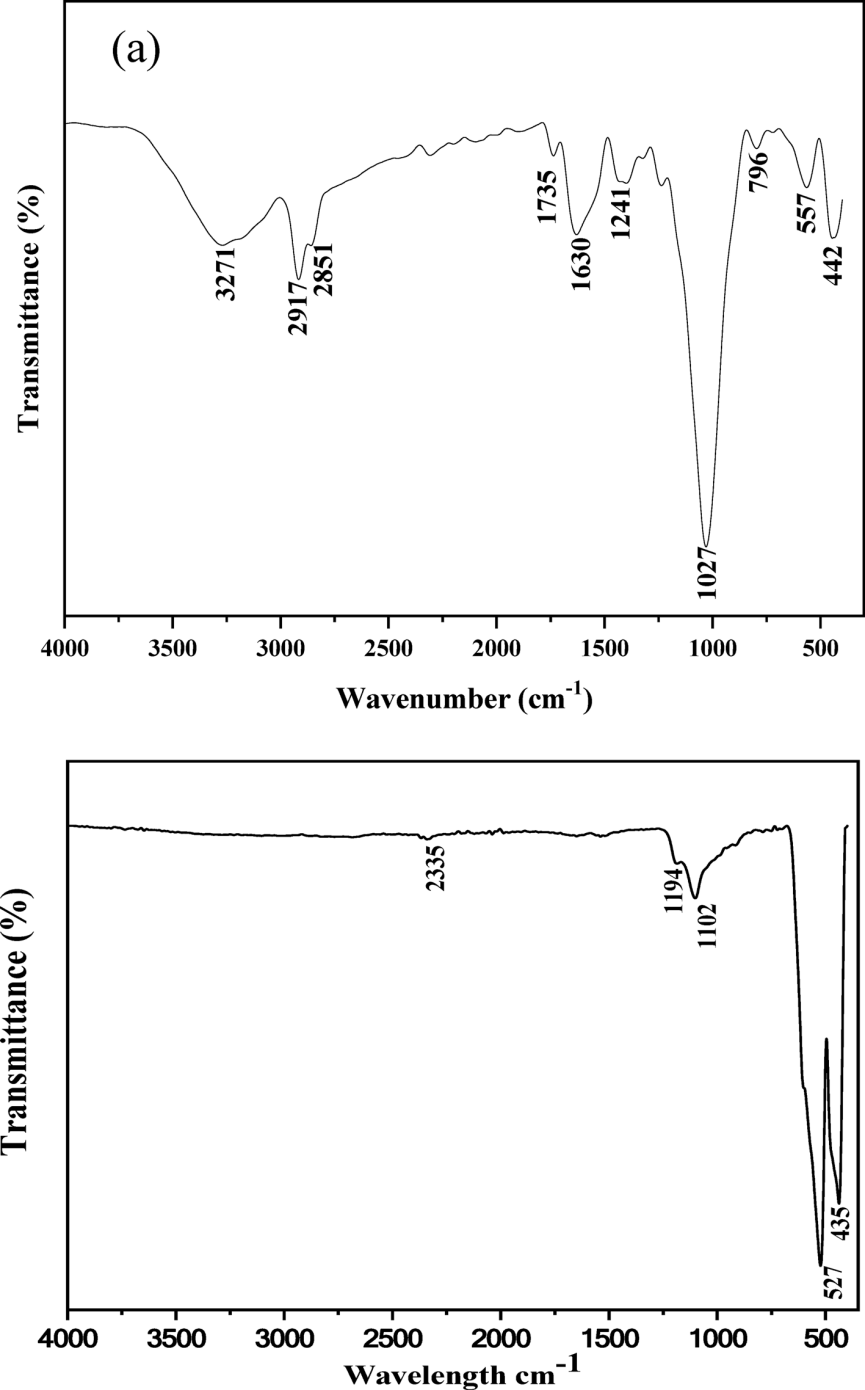
FTIR spectra of **a** extract of *Sorghum bicolor* leaves and **b** α-Fe_2_O_3_ NPs

The FTIR spectrum of *Sorghum bicolor* leaf represented in Fig. 3a shows broad and sharp peaks around 3271 and 1630 cm^−1^, which are associated with the O-H stretching and bending vibrations, respectively. These peaks are associated with the water molecules from the raw cellulose [41–43]. Symmetric and asymmetric C–H stretching vibrations of unsaturated SP^2^ hybridised hydrocarbon were identified around 2917 and 2851 cm-^1^, respectively [44]. The band around 1735 cm-^1^ may be ascribed to the presence of carbonyl C = O stretch of hemicellulose [45–48], while the band at 1027 cm-1 reveals the C-O stretch vibrations [49]. These peaks are indicative of the *Sorghum* fibre’s hydrophilic properties. The FTIR spectrum of the biosynthesized α-Fe_2_O_3_ NPs is presented in Fig. 3b. The peaks at 527 and 435 cm^− 1^ could be ascribed to the Fe–O stretching and Fe–O bending vibrations in the crystalline lattice of α-Fe_2_O_3_ [50, 51]. Bands between 400 and 600 cm^− 1^ are often ascribed to the stretching vibration mode that is associated with the metal-oxygen. The peak around 2335 cm^− 1^ is related to the asymmetric stretching vibration of the O–C–O group, likely due to the presence of absorbed CO_2_ on the nanoparticles [52]. This may be confirmed by the appearance of the band around 1102 cm^− 1^ [53]. The absence of the bands observed around 3271 and 1630 cm^− 1^, which are attributed to the O–H vibrations from the plant extract, as well as the C–H vibrational bands are indicative of the disappearance of the phytochemical components of the materials at the high calcination temperature.

### X-ray diffraction analysis (XRD)

**Fig. 4:**
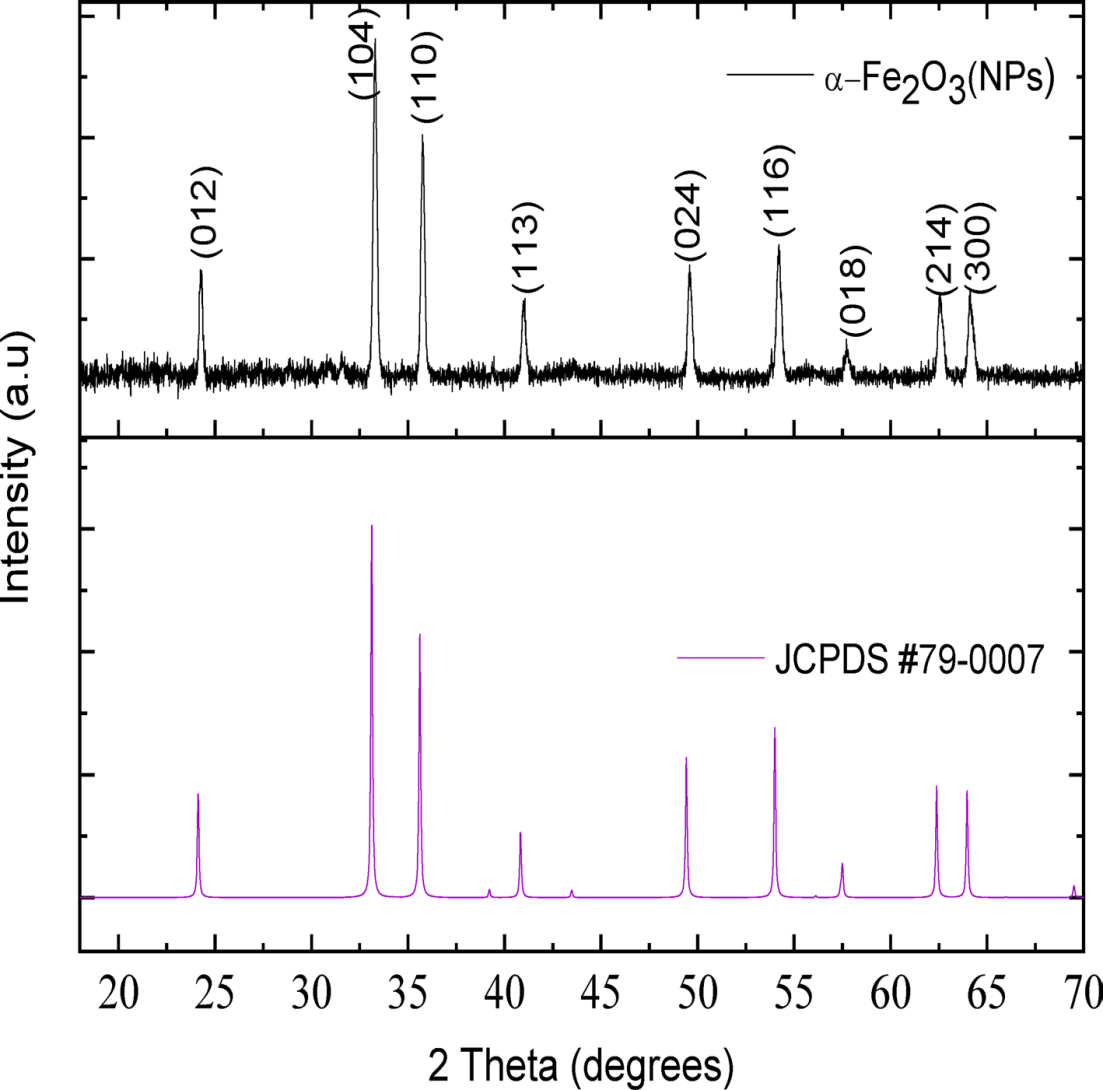
XRD pattern of α-Fe_2_O_3_ NPs mediated by aqueous extract of *Sorghum bicolor* leaves

The XRD pattern of *Sorghum bicolor* leaf extract-mediated iron oxide nanoparticles is shown in Fig. 4. It shows diffraction peaks at 2θ values of (24.26º), (33.37º), (35.74º), (41.02º), (49.61º), (54.27º), (57.68º), (62.65º), and (64.20º), corresponding to the ((012), (104), (110), (113), (024), (116), (018), (214), and (300) planes of the rhombohedral hematite phase of nanoparticles (α-Fe_2_O_3_) (JCPDS 79-0007) [54]. The absence of additional peaks in the XRD pattern confirms the high purity of the green-synthesized α-Fe_2_O_3_ nanoparticles. Furthermore, the intensity and sharpness of the peaks indicated that the nanoparticles were crystalline [55]. Using the Debye-Scherrer formula in Eq. 4, the crystallite size of the nanoparticles was estimated from the most predominant (104) plane of the synthesized hematite nanoparticles to be approximately 46.8 nm.

4 $$D = \frac{{K{{\uplambda }}}}{{\beta Cos~\theta }}$$

where, λ is the wavelength of the X-ray (1.5406 Å), K represents the shape factor (0.9), θ represents the diffraction angle given in degrees, and β is the full width at half maxima (FWHM) of the peaks of interest [56].

Rietveld refinement of the XRD data was performed using X’Per Highscore plus software to extract the structural details (Table 2). The α-Fe_2_O_3_ nanoparticles showed large grain size and good crystallinity, but low porosity. The large grain size could be due to grain growth mechanisms, which often take place at high temperatures via diffusion and coalescence [57]. This is also responsible for the low porosity of the sample. A previous study showed that increasing hematite’s calcination temperature results in a consistent increase in the crystal density of the phase. This was an indication of a decrease in the degree of disorder in the crystal lattice and growth of the binding energy of lattice oxygen [58].

**Table 2:** Structural parameters of α-Fe_2_O_3_ obtained from Rietveld refinement

Sample	Grainsize (µm)	% Crystallinity	% Porosity	Surface area (m^2^/g)
α-Fe_2_O_3_	2.791	89.94	0.134	0.0126

### UV-Vis spectra analysis

**Fig. 5:**
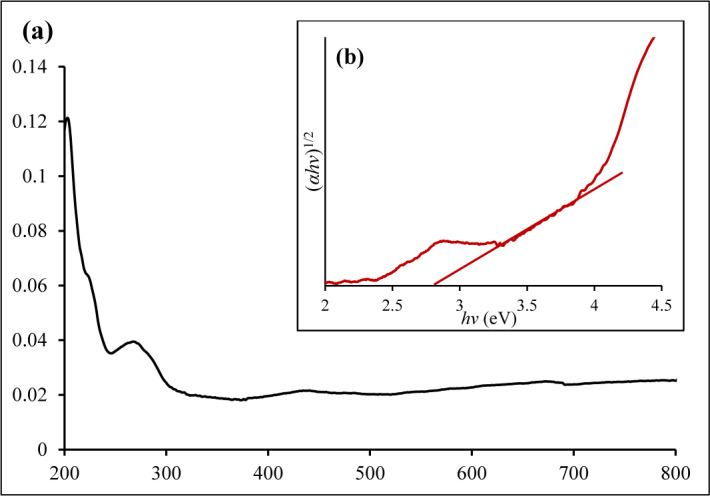
**a** The UV-visible spectrum and **b** Tauc plots of the α-Fe_2_O_3_ NPs prepared from the aqueous extract of *Sorghum bicolor* leaves

Figure 5a and b present the UV-visible absorption spectrum and the corresponding Tauc plots of the as-synthesized α-Fe_2_O_3_ NPs, respectively. As illustrated in Fig. 5a, the NPs exhibit an absorption peak around 265 nm and a weak absorption around 435 nm that broadens to 600 nm within the visible light region. A similar absorption band has been observed for α-Fe_2_O_3_ nanoparticles synthesized using various plant extracts, including the root extract of *Spondias dulcis* [59], and *Phoenix dactylifera* leaf extract [60]. The band gap energy of the α-Fe_2_O_3_ nanoparticles was determined using a Tauc plot (inset of Fig. 5b) and was obtained as 2.8 eV. This aligns closely with the previously reported value of 2.75 eV for α-Fe_2_O_3_ synthesized using henna extract [61], and 2.80 eV reported by Anit et al. [62]. The band gap energy of α-Fe_2_O_3_ can vary depending on factors such as synthesis method or introduction of foreign materials such as dopants.

### Morphological studies

**Fig. 6:**
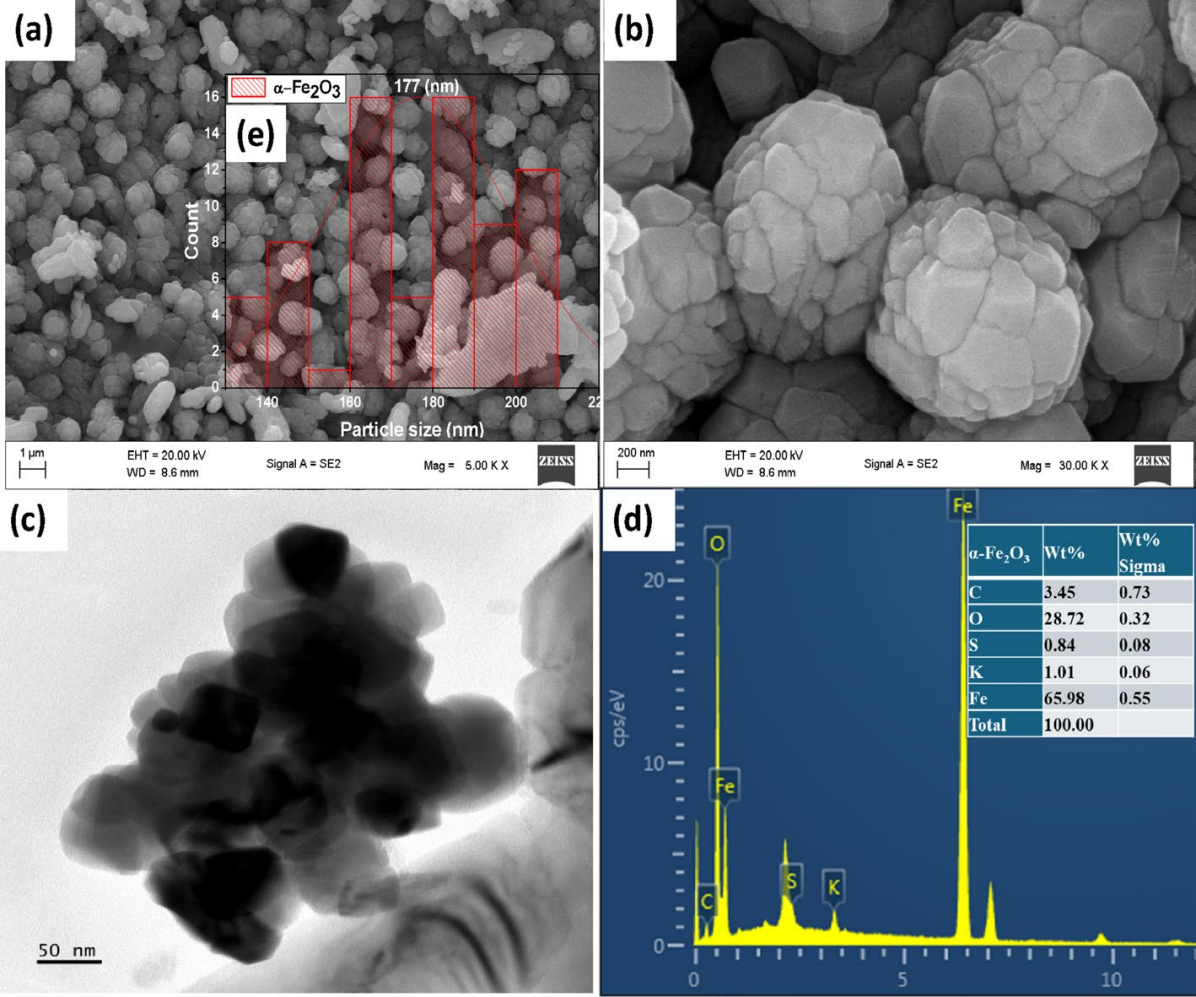
SEM micrograph at **a** low, **b** high magnification, **c** TEM images, **d** EDX spectrum, and inset (e) particle size distribution histogram of α-Fe_2_O_3_ nanoparticles synthesized using aqueous extract of *Sorghum bicolor* leaf

The morphological properties of the α-Fe_2_O_3_ nanoparticles were determined using both SEM and TEM characterization techniques. The SEM analysis showed that the NPs exhibited a distinctly spherical shape (Fig. 6a), while the higher magnification image (Fig. 6b) confirmed well-aggregated particles that are clustered together to form a uniform structure. This aggregation may be due to the interaction of the charges present on the surface of the nanoparticles, which has been reported to increase as the size of the particles within the nanometric regime [63]. The high-temperature calcination reaction of the nanoparticles has also been reported to increase the surface reactivity, thereby enhancing aggregation [64]. Kaur et al. [65] reported the green synthesis of nanoparticles using the extract of *Carica papaya*, and attributed the aggregation of NPs to the presence of an excess of H^+^ ions from the H_2_O present on the surface of the nanoparticles [65]. However, the capping role of the phytocomponent obtained from the plant can protect the nanoparticles from agglomeration [66]. The particle size distribution histogram obtained from the SEM images shows that the agglomerated particles have an average size of 177 nm. This large size could be ascribed to fusion of the individual particles to form large spherical agglomerates. The TEM analysis (Fig. 6c) also showed highly agglomerated spherical morphology with no distinct edge. Green synthesized nanoparticles can give rise to varying sizes and morphologies such as squares, triangles, and spheres [67]. This variation has been attributed to the effect of the capping agent derived from compounds found in the leaf extracts and the synthesis temperature. Figure 6d presents the EDX spectrum of the α-Fe_2_O_3_ nanoparticles, which shows the elemental composition and confirms that iron (Fe) and oxygen (O) are the major components of the nanoparticles. The presence of potassium (K) and sulfur (S) could be from the plant extract, while the carbon (C) peak found could be ascribed to the emission from the carbon ribbon used during the analysis.

### Anticancer evaluation of α-Fe_2_O_3_-NPs

**Fig. 7:**
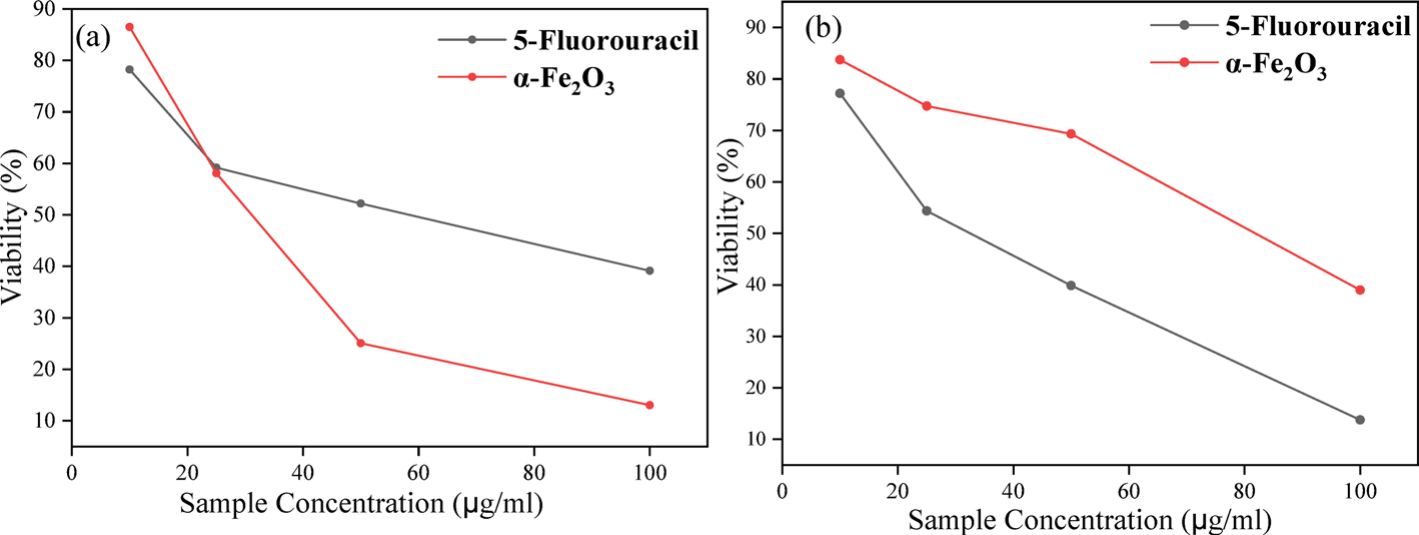
Percentage viability of **a** HeLa cell line and **b** HEK293 cell line at different concentrations of 5-Fu and α-Fe_2_O_3_

Table 3 presents the cytotoxic effects of αFe_2_O_3_ nanoparticles and the standard chemotherapeutic agent, 5-fluorouracil (5-FU), on HeLa and HEK293 cell lines at different concentrations (10, 25, 50, and 100 µg/mL). The prepared α-Fe_2_O_3_-NPs demonstrated cytotoxic effects against HeLa and HEK293 cell lines, though their potency was lower than that of the standard chemotherapeutic agent 5-FU. The percentage viability of Hela and HEK 293 cell lines at different concentrations of 5-Fu and α-Fe_2_O_3_ is presented in Fig. 7. The viability of HeLa cells decreased in a dose-dependent manner upon exposure to α-Fe_2_O_3_-NPs, dropping from 86.49% at 10 µg/mL to 13.03% at 100 µg/mL, with an IC_50_ of 26.31 µg/mL. In comparison, 5-FU exhibited greater cytotoxicity, reducing HeLa cell viability from 78.24% at 10 µg/mL to 39.15% at 100 µg/mL, with a lower IC_50_ of 17.46 µg/mL. The higher IC_50_ of α-Fe_2_O_3_-NPs suggests that a greater concentration is required to achieve 50% inhibition, indicating lower potency compared to 5-FU. However, the ability of α-Fe_2_O_3_-NPs to reduce cancer cell viability highlights their potential as anticancer agents, possibly through mechanisms such as oxidative stress induction, apoptosis activation, and disruption of cellular iron homeostasis [68].

Interestingly, α-Fe_2_O_3_-NPs exhibited significantly lower toxicity toward HEK293 normal cells compared to 5-FU. While 5-FU drastically reduced HEK293 cell viability from 77.19% at 10 µg/mL to 13.76% at 100 µg/mL, with a very low IC_50_ of 6.71 µg/mL, α-Fe_2_O_3_-NPs displayed a more gradual cytotoxic effect, maintaining higher viability across all tested concentrations and yielding an IC_50_ of 69.19 µg/mL. This substantial difference suggests that α-Fe_2_O_3_-NPs exhibit selective toxicity, preferentially targeting cancer cells while sparing normal cells. The observed selectivity may be attributed to differences in nanoparticle uptake, metabolic activity, and oxidative stress handling between cancerous and non-cancerous cells. Cancer cells typically exhibit increased nanoparticle internalization due to altered endocytotic pathways, as well as heightened susceptibility to ROS-mediated damage due to their already elevated oxidative stress levels [69]. In contrast, normal cells possess more robust antioxidant defense mechanisms, which may account for their increased resistance to α-Fe_2_O_3_-NP-induced cytotoxicity [70].

**Table 3:** HeLa and HEK293 cell line viability (%) at varying α-Fe_2_O_3_-NPs concentrations

Sample	10 µg/mL^− 1^	25 µg/mL^− 1^	50 µg/mL^− 1^	100 µg/mL^− 1^	IC_50_
HeLa					
5-FU	78.24 ± 0.015	59.16 ± 0.048	52.21 ± 0.019	39.15 ± 0.031	17.46
α-Fe_2_O_3_	86.49 ± 0.119	58.06 ± 0.087	25.89 ± 0.029	13.03 ± 0.050	26.31
HEK293					
5-FU	77.19 ± 0.035	54.36 ± 0.014	39.87 ± 0.033	13.76 ± 0.025	6.71
α-Fe_2_O_3_	83.73 ± 0.071	74.74 ± 0.040	69.32 ± 0.017	39.00 ± 0.072	69.19

The cytotoxic effects of α-Fe_2_O_3_-NPs in HeLa cells can be linked to several biological mechanisms, including ROS generation, apoptosis induction, and ferroptosis. The enhanced oxidative stress caused by α-Fe_2_O_3_-NPs likely disrupts mitochondrial function, leading to DNA damage and activation of apoptotic pathways [71, 72]. Additionally, α-Fe_2_O_3_-NPs may interfere with iron metabolism, triggering ferroptosis, a non-apoptotic form of programmed cell death characterized by lipid peroxidation and membrane damage [69]. The progressive decline in cell viability at increasing α-Fe_2_O_3_-NP concentrations supports this hypothesis, as cancer cells rely heavily on iron homeostasis for survival and proliferation. The involvement of apoptosis is further suggested by the potential modulation of pro-apoptotic and anti-apoptotic proteins, such as Bax and Bcl-2, which regulate mitochondrial membrane integrity and caspase activation [70].

The differential cytotoxic effects observed in HeLa and HEK293 cells highlight the potential of α-Fe_2_O_3_-NPs as selective anticancer agents. Compared to 5-FU, α-Fe_2_O_3_-NPs demonstrate a more favorable safety profile by causing significantly less damage to normal cells. This suggests that α-Fe_2_O_3_-NPs could serve as a nanomedicine-based alternative to conventional chemotherapy, potentially reducing systemic toxicity and adverse side effects such as myelosuppression and gastrointestinal disturbances [68]. Additionally, the selective cytotoxicity of α-Fe_2_O_3_-NPs presents an opportunity for their use in combination therapy, where they could enhance the efficacy of traditional chemotherapeutics while mitigating their toxic effects on healthy tissues [73]. Hence, the findings of this study support the growing interest in metal-based nanoparticles for cancer therapy, particularly due to their unique physicochemical properties that enable targeted interactions with cancer cells [74]. Table 4 presents the evaluation of the anticancer potential of α-Fe_2_O_3_-NPs synthesized from *Sorghum bicolor* extract in comparison to other iron oxides, composites, and α-Fe_2_O_3_-NPs reported in the literature.

**Table 4:** Evaluation of the anticancer potential of α-Fe_2_O_3_ nanoparticles synthesized from *Sorghum bicolor* extract in comparison to other α-Fe_2_O_3_ nanoparticles documented in the literature

Nanoparticle	Concentration (µg/mL)	IC50 (µg/mL)	% Cell viability	Ref
FeO	100	–	87.02	[68]
TR-FeO	140	–	76.09	[68]
Fe_2_O_3_	100	40.42	46.11	[75]
Fe_2_O_3_@ CuO	100	21.13	35.42	[75]
FeO_2_	100	–	50	[76]
α-Fe_2_O_3_	104.6	50	50	[69]
α-Fe_2_O_3_	100	26.31	13.03	This study
α-Fe_2_O_3_	100	85.19	39.00	This study

The data are presented as mean cell viability (%) ± standard deviation (*n* = 3), with fluorouracil serving as the reference standard.

### Antioxidant activity analysis of α-Fe_2_O_3_-NPs

**Fig. 8:**
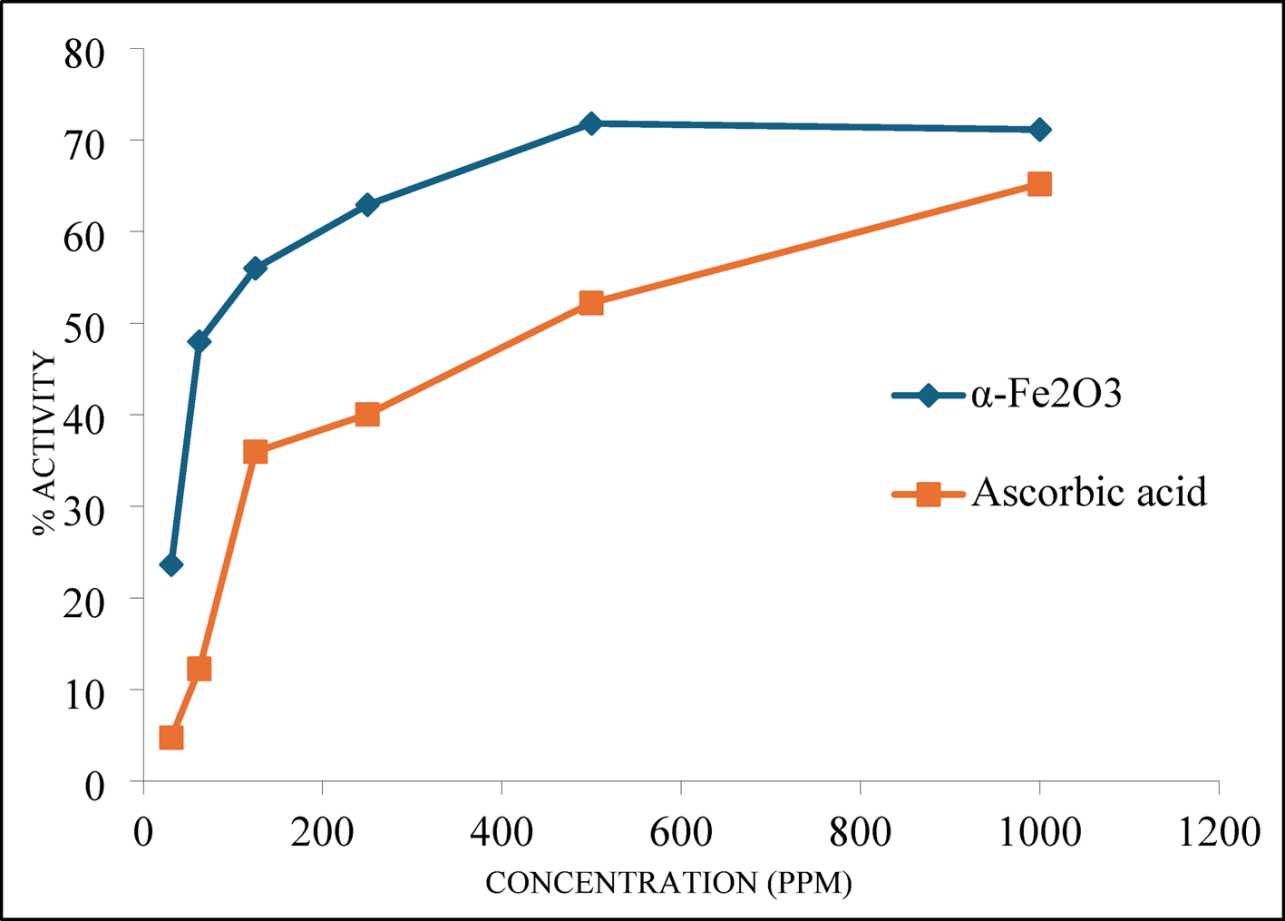
Radical scavenging activity (%) of α-Fe_2_O_3_-NPs and ascorbic acid at various concentration

Table 5; Fig. 8 present the antioxidant activity of α-Fe_2_O_3_-NPs against ascorbic acid at varying concentrations (31.25–1000 ppm). The inhibition percentage increased in a concentration-dependent manner for both α-Fe_2_O_3_-NPs and ascorbic acid. However, ascorbic acid demonstrated superior antioxidant efficacy. At doses of 31.25 and 62.5 ppm, α-Fe_2_O_3_-NPs exhibited inhibition rates of 23.63% and 47.99%, respectively, while ascorbic acid showed 4.73% and 12.26% inhibition at the same concentrations. However, the IC_50_ value of α-Fe_2_O_3_-NPs (168.83 ppm) was much higher than ascorbic acid (3.48 ppm), indicating that a greater concentration α-Fe_2_O_3_-NPs was needed to achieve 50% radical inhibition. Despite the higher IC_50_, the strong antioxidant activity observed in α-Fe_2_O_3_-NPs at higher concentrations suggests their potential role in combating oxidative stress, which is involved in various pathological conditions, such as cancer, inflammatory diseases, and neurodegenerative disorders.

**Table 5:** Antioxidant activity (%) of α-Fe_2_O_3_-NPs

Samples			Conc. (ppm)				IC_50_
	31.25	62.5	125	250	500	1000	
Ascorbic acid	4.73 ± 0.02	12.26 ± 0.06	35.95 ± 0.04	40.04 ± 0.01	52.21 ± 0.00	65.20 ± 0.02	3.48
α-Fe_2_O_3_-NPs	23.63 ± 0.01	47.99 ± 0.11	55.99 ± 0.06	62.91 ± 0.01	71.81 ± 0.01	71.14 ± 0.13	168.83

Values are presented as the mean inhibition percentage (%) ± deviation (*n* = 3), with ascorbic acid serving as the standard

The antioxidant capacity of α-Fe_2_O_3_-NPs can be attributed to several underlying mechanisms. One key pathway involves their ability to neutralize ROS and reactive nitrogen species (RNS) through electron donation, thereby preventing oxidative damage to vital biomolecules such as lipids, proteins, and DNA [77]. This redox activity is likely facilitated by the iron component of α-Fe_2_O_3_-NPs, which can participate in Fenton and Haber-Weiss reactions, influencing cellular oxidative balance. While this redox cycling can be beneficial in scavenging free radicals, its effects can vary depending on the biological microenvironment, potentially shifting between protective and pro-oxidant roles [78]. Additionally, α-Fe_2_O_3_-NPs may enhance endogenous antioxidant defense systems by upregulating key enzymes such as superoxide dismutase (SOD), catalase (CAT), and glutathione peroxidase (GPx) [79]. These enzymes play critical roles in mitigating oxidative stress and maintaining cellular homeostasis, which could contribute to the overall protective effects observed with α-Fe_2_O_3_-NPs.

Beyond their direct antioxidant effects, α-Fe_2_O_3_-NPs may have significant therapeutic implications. The ability to modulate oxidative stress suggests potential applications in adjunctive cancer therapy, where α-Fe_2_O_3_-NPs could help protect normal cells from chemotherapy-induced oxidative damage while still exerting selective cytotoxicity on cancer cells [80]. Similarly, α-Fe_2_O_3_-NPs could be valuable in managing neurodegenerative diseases such as Alzheimer’s and Parkinson’s, where oxidative stress is a key driver of neuronal damage and disease progression [80]. Their capacity to reduce oxidative burden could also be beneficial in tissue regeneration and wound healing, where excessive oxidative stress impairs cellular repair mechanisms. Furthermore, their anti-inflammatory potential may contribute to reducing oxidative damage in chronic inflammatory conditions. Thus, the result of the present study indicates that α-Fe_2_O_3_-NPs possess strong antioxidant properties that could be leveraged for various biomedical applications [81, 82]. Their ability to scavenge free radicals, modulate redox balance, and enhance endogenous defense mechanisms position them as promising candidates for therapeutic interventions aimed at reducing oxidative stress-related damage. Table 6 presents the evaluation of the antioxidant potential of α-Fe_2_O_3_ nanoparticles prepared from *Sorghum bicolor* extract in comparison to other biosynthesized iron oxide nanoparticles documented in the literature. NPs, can exhibit both antioxidant and anticancer activities, but these effects often operate through distinct and sometimes opposing mechanisms. Interestingly, NPs with lower antioxidant activity may demonstrate higher anticancer efficacy due to their ability to induce oxidative stress selectively in cancer cells. The mechanism of α-Fe_2_O_3_ NPs, with antioxidant properties scavenge free radicals, thereby reducing cellular stress and preventing damage to DNA, proteins, and lipids.

**Table 6:** Evaluation of the antioxidant potential of α-Fe_2_O_3_ nanoparticles synthesized from *Sorghum bicolor* extract in comparison to other reported iron oxide nanoparticles

Material	Plant extract	IC_50_	Ref
Fe_2_O_3_	*Pheonix dactylifera*	26.4 mg/g	[83]
Fe_2_O_3_	*Ficus carcia*	12.12 mg/mL	[84]
Fe_x_O_y_-NPs	*Pheonix dactylifera* L.	2.12 mg/mL	[85]
α-Fe_2_O_3_	Ginger extract	312.11 µg/mL	[86]
IO-NPS	*Laurus nobilis*	122.57 µg/mL	[87]
IO-NPS	*Sageretia thea*	33.85 µg/mL	[70]
α-Fe_2_O_3_	*Sorghum bicolor*	168.83 ppm	This study

## Conclusion

In conclusion, the green synthesis of hematite nanoparticles (α-Fe_2_O_3_ NPs) using *Sorghum bicolo*r leaf extracts provides an environmentally friendly and cost-effective approach to nanoparticles synthesis. The synthesized NPs exhibited a crystalline rhombohedral hematite phase with spherical morphology and elemental composition confirming Fe and O as major components. The biological evaluations demonstrated selective cytotoxicity towards HeLa cancer cells while sparing normal HEK293 cells, supporting the role of iron homeostasis in cancer cell survival. Although the potency was lower than the standard chemotherapeutic agent 5-FU, the concentration-dependent reduction in cell viability suggests potential biomedical applications. Overall, this study highlights the promising use of *Sorghum bicolor*-mediated α-Fe_2_O_3_ NPs in nanomedicine, warranting further exploration of their therapeutic potential.

## Data Availability

“Data is provided within the manuscript”.
