# Correction: Biogenic α-Fe_2_O_3_ nanoparticles from *Sorghum bicolor* leaf extracts and assessment of the anticancer and antioxidant properties

**DOI:** 10.1186/s11671-025-04344-1

**Published:** 2025-09-10

**Authors:** Lerato D. Msimango, Mercy C. Ogwuegbu, Doctor M. N. Mthiyane, Damian C. Onwudiwe

**Affiliations:** 1 Department of Animal Science, Faculty of Natural and Agricultural Science, North-West University, Mmabatho, 2035 South Africa; 2 Food Security and Safety Focus Area, Faculty of Natural and Agricultural Sciences, North-West University, Mmabatho, 2735 South Africa; 3 Department of Animal Science, Faculty of Agriculture, University of Nigeria, Nsukka, 410001 Enugu State Nigeria; 4 Materials Science Innovation and Modelling (MaSIM), Faculty of Natural and Agricultural Sciences, North-West University, Mmabatho, South Africa; 5 Department of Chemistry, School of Physical and Chemical Sciences, Faculty of Natural and Agricultural Science, North-West University (Mafikeng Campus), Private Bag X2046, Mmabatho, South Africa

**Correction to: Discover Nano (2025) 20:113** 10.1186/s11671-025-04281-z

Following the publication of the original article [1], it was noted that the author’s name Doctor M.N Mthiyane was incorrectly written as M.N Mthiyane. The original article [1] has been corrected.
